# Prenatal diagnosis following preimplantation genetic testing for monogenic conditions: a single centre record linkage study

**DOI:** 10.1007/s10815-024-03346-9

**Published:** 2025-01-24

**Authors:** Alice Poulton, Melody Menezes, Tristan Hardy, Sharon Lewis, Lisa Hui

**Affiliations:** 1 Monash IVF Group LTD, VIC Clayton, Australia; 2 University of Melbourne, Parkville, Australia VIC; 3 Murdoch Children’s Research Institute, Parkville, Australia VIC; 4 Victorian Clinical Genetics Service, Parkville, Australia VIC; 5 Mercy Hospital for Women, Heidelberg, Australia VIC; 6 The Northern Hospital, Epping, Australia VIC

**Keywords:** Preimplantation genetic testing, Prenatal diagnosis, PGT-M, Karyomapping, Single gene disorder, 24-chromosome aneuploidy screening

## Abstract

**Purpose:**

Professional bodies currently advise all pregnant individuals undertake confirmatory prenatal diagnostic testing following preimplantation genetic testing for monogenic conditions (PGT-M). We aimed to ascertain the uptake of prenatal diagnostic testing following PGT-M in a large single-centre population.

**Methods:**

This observational linkage study was undertaken using routinely collected outcome data from PGT-M cycles performed at one of Australia’s largest PGT-M providers and a statewide dataset of all prenatal samples undergoing cytogenetic analysis in Victoria, Australia, between 2015 and 2022.

**Results:**

During the study period, there were 176 clinical pregnancies following the transfer of a PGT-M-tested embryo in 132 patients. Eleven patients undertook confirmatory prenatal diagnostic testing in 12 pregnancies, representing a confirmatory testing rate of 8.3% [95% CI: 4.7–14.3%] per patient and 6.8% [95% CI: 3.9–11.5%] per pregnancy. The 176 clinical pregnancies resulted in 154 (87.5%) live births and pregnancies ongoing at the time of reporting, 21 (11.9%) pregnancy losses ≤ 20 weeks gestation, and 1 (0.6%) stillbirth.

**Conclusions:**

Most patients who conceive following the transfer of a PGT-M-tested embryo do not undertake confirmatory prenatal diagnostic testing. The low uptake of confirmatory testing raises important considerations for genetic counselling for PGT-M and the acceptability of current clinical practice recommendations.

## Introduction

Pre-implantation genetic testing for monogenic conditions (PGT-M) is a technique used to characterise the genetic status of cells biopsied from embryos created in vitro [1]. This technique allows the selection and transfer of unaffected embryos to the uterus and may be used as a reproductive option for couples with an increased chance of having a child with a monogenic condition. For many prospective parents, PGT-M is their preferred reproductive option as it allows for the conception of a biologically related child, while significantly reducing the chance of an affected pregnancy. This in turn lessens the need to consider options that follow prenatal diagnosis, such as preparing for the birth of a child with the monogenic condition, or considering termination of pregnancy [2, 3].

Advances in molecular genetics (including the development of multiplex polymerase chain reaction protocols, whole genome amplification methods, and genome-wide haplotyping techniques) have been quickly translated to PGT-M, significantly increasing its resolution. However, despite these advances, misdiagnosis remains a limitation of the technology due to various technical, biological, and human factors [4]. In 2009, the European Society of Human Reproduction and Embryology (ESHRE) consortium published data on PGT-M cycles and deliveries from 1997 to 2009. They reported 12 misdiagnoses and adverse outcomes out of 2538 cycles using PCR-based analysis, resulting in a misdiagnosis rate of 0.07% [5]. Although data on misdiagnoses following genomic-haplotyping is currently unavailable, this testing approach is expected to be more reliable, due to the detection of a higher density of informative markers around the gene of interest — improving the detection of allele drop out and recombination events.

Despite the low occurrence of misdiagnosis, some centres have faced litigation due to such errors. In some instances, claims of medical negligence have focused on a lack of informed consent, with patients alleging they were not adequately informed about the limitations of the technology [6]. Professional bodies, including the Society for Assisted Reproductive Technology and American Society For Reproductive Medicine, recommend patients be offered prenatal diagnosis for confirmation of genetic status in a pregnancy conceived after a PGT-M cycle [7–9]. However, recent ESHRE guidelines (2020) only recommend prenatal diagnosis in specific scenarios, such as cases involving de novo variants, or germline mosaicism. The variability in existing guidelines highlights evolving perspectives on the importance of confirmatory testing following PGT-M.

Surveys of clinical practice indicate nearly all clinics follow recommendations and offer confirmatory prenatal diagnostic testing [4]. However, the uptake of prenatal diagnosis appears variable. A 2022 study investigating uptake of prenatal diagnosis following PGT-M in Boston reported only 12% of patients (*n* = 142) underwent confirmatory prenatal diagnostic testing [10]. Similarly, low rates of confirmatory testing are observed following PGT testing for aneuploidy, with Kimelman et al. reporting only 6% of patients (*n* = 68) had confirmatory testing following PGT-A in Chicago [7]. Conversely, a 2022 Danish survey study reported that 44.8% of respondents (*n* = 172) opted for CVS following PGT-M.

Several factors may influence a patient’s decision to undergoing prenatal diagnosis following PGT-M, including the perceived risk of misdiagnosis, risk of procedure-related miscarriage, the potential for an ultrasound diagnosis to provide an alternative to invasive testing, and the specific characteristics of the condition (such as severity and time of onset) [11, 12].

There are methodological challenges in obtaining complete ascertainment of prenatal diagnostic testing after PGT-M. Obstetric care is typically with a different provider than the fertility specialist performing the IVF and PGT-M. Additionally, prenatal diagnosis is not part of the mandatory outcome collection stipulated by the Reproductive Technology Accreditation Committee (RTAC) code of practice in Australia [13]. Given the paucity of literature on prenatal diagnosis after PGT-M, we aimed to perform an individual patient linkage study to ascertain uptake of prenatal diagnostic testing following PGT-M in an Australian population. We also examined differences in uptake of confirmatory prenatal diagnostic testing according to disease characteristics.

## Methods

We performed individual patient record linkage between routinely collected data from PGT-M cycles performed at Monash IVF, a private IVF centre in Victoria, Australia, and a statewide data collection of all cytogenetic prenatal diagnosis. We linked data collected over an 8-year period between 2015 and 2022.

We also systematically categorised monogenic indications within the PGT-M cohort according to disease characteristics, so differences in uptake of confirmatory testing could be analysed across categories. 

### Study setting

Victoria has approximately 76,000 births annually [14]. Chorionic villus sampling (CVS) and amniocentesis are available in both public and private settings [15]. There are no direct costs to patients if they access care as a public patient in a public hospital. Patients who elect to have private obstetric care incur out-of-pocket expenses. The cost of CVS and amniocentesis procedures ranges between $600 and 800 AUD [16]. The cost of monogenic analysis of the sample can vary based on the complexity and nature of the analysis and can range from $500 to 2000 AUD [17]. These costs are partially subsidised by government rebates.

Over the study period, a mean of 1606 procedures (CVS or amniocentesis) (range 1388–1957) are performed annually in the state, with a mean of 166 procedures (range 134–193) performed annually for monogenic indications, including pregnancies with and without PGT-M [18].

There are approximately 200 PGT-M cycles each year in Victoria. At the IVF clinic, out-of-pocket expenses are incurred for PGT-M. An IVF/ICSI cycle is approximately $6000 AUD, while embryo biopsy fees are approximately $600 AUD per cycle and testing fees are approximately $800 AUD per embryo. As of 2020, PGT-M fees are partially subsidised by government rebates, which return up to $1800 AUD for the testing of three embryos. There are currently no government rebates for PGT-A.

### Datasets 

#### Victorian prenatal diagnosis dataset

The Victorian Prenatal Diagnosis Dataset (VPDD) is a statewide data collection of prenatal cytogenetic testing, including chromosomal microarray, G-banded karyotypes, and rapid aneuploidy tests [19]. Indication for the diagnostic procedure (amniocentesis or CVS) is provided, including those performed due to risk of a fetal monogenic condition, prenatal screening results, or ultrasound phenotype. Information on indication for testing subsequently enabled us to identify the primary reason for prenatal diagnostic testing. Only the results of concurrent chromosomal analysis are available.

There is currently no central data repository capturing the outcomes of prenatal monogenic testing. However, in Victoria, it is routine for all prenatal samples undergoing monogenic analysis to also have a molecular karyotype. Therefore, ascertainment of these cases through the database was assumed to be complete.

The VPDD includes the following information on each case: first three letters of first name, first three letters of surname, date of birth, date of diagnostic procedure, gestation at time of diagnostic procedure, type of diagnostic procedure, indication for testing, and results of cytogenetic analysis.

## Monash IVF PGT-M cohort

Data were collected on clinical pregnancies following the transfer of PGT-M tested embryos between January 2015 and December 2022 at Monash IVF, a private IVF centre in Victoria, Australia.

Collected data included patient first name, surname, date of birth, monogenic indication, date of embryo transfer, confirmation of pregnancy status, PGT monogenic result, PGT chromosomal result, and pregnancy outcome.

Postnatal testing is not routinely performed for all monogenic indications, particularly those with adult age of onset. Postnatal testing outcomes were subsequently unavailable, preventing us from ascertaining a misdiagnosis rate. As prenatal testing uptake was limited and comprehensive outcome data are not centrally available, we were also unable to determine a misdiagnosis rate using prenatal testing outcomes. A misdiagnosis rate was therefore unable to be ascertained for this cohort.

### Clinical protocol for PGT-M cohort

The following methodology describes standard clinical procedures associated with PGT-M at the IVF centre over the study period.

All patients receive pre-test counselling from their fertility specialist, clinical geneticist and/or genetic counsellor. Pre-test counselling involves review of the monogenic indication and discussion surrounding the timing, process, limitations, and costs of PGT. The possibility of misdiagnosis is discussed, and confirmatory prenatal diagnosis in any ensuing pregnancy is recommended. Prospective patients are advised that PGT-M is between 95 and 98% accurate.

Ovarian stimulation and oocyte retrieval procedures follow published protocols [20, 21]. All oocytes are fertilised using intracytoplasmic sperm injection (ICSI). Following ICSI, embryos are assessed, developmentally classified and quality graded as previously described [21, 22]. Embryos are considered suitable for biopsy on day 5 if they contain a clearly defined inner cell mass and a suitable number of healthy trophectoderm cells. Approximately five trophectoderm cells are biopsied for PGT-M using a combination of laser and mechanical biopsy techniques. Whole genome amplification is performed on biopsy samples using the RepliG Single Cell Kit (Qiagen, the Netherlands). Karyomapping is performed at Monash IVF genetics laboratory as previously described [23]. The use of karyomapping enables simultaneous haplotype phasing and 24-chromosome screening (known as preimplantation genetic testing for aneuploidy, or PGT-A).

Frozen-thawed embryos are transferred on day 5 of either a natural or hormone replacement cycle [20]. To confirm biochemical pregnancy status, human chorionic gonadotrophic (hCG) testing is undertaken ~ 14 days post embryo transfer. To confirm clinical pregnancy status, a viability ultrasound is performed between 7 and 9 weeks. Following confirmation of pregnancy, patients are referred by their fertility specialist for ongoing obstetric care.

Treating obstetricians provide birth outcomes to the fertility centre in compliance with the licensing requirements of the RTAC. The RTAC Code of Practice mandates reporting of all pregnancy outcomes to the Australian and New Zealand Assisted Reproduction Database (ANZARD) for clinical quality monitoring [13].

### Linkage, analysis and categorisation according to monogenic indication

#### Data linkage

**Fig. 1:**
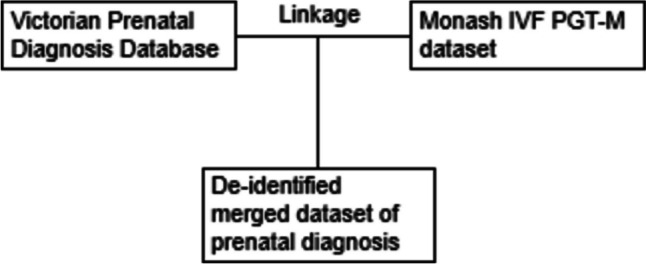
Flow diagram of linkage

Automated probabilistic linkage analysis was undertaken using LinkageWiz data matching software (LinkageWiz Inc., Adelaide) to track pre- and post-conception genetic testing pathways (Fig. 1). LinkageWiz is a propriety software tool used for probabilistic data matching between large datasets. Date of birth, first three letters of the first and last name, and monogenic indication were used as identifiers to link the datasets. All potential data matches were reviewed manually, and the date of embryo transfer and date of prenatal diagnostic procedure were used to confirm if testing had taken place during the same pregnancy. This enabled the assessment of the utilisation of confirmatory testing in Victoria, and the calculation of the uptake of prenatal diagnostic testing following the use of PGT-M.

##### Descriptive caption

 A flow chart illustrating the process of linking two datasets: the Victorian Prenatal Diagnosis Database and the Monash IVF PGT-M dataset. The linkage results in a de-identified merged dataset, which consolidates prenatal diagnosis information for further analysis. 

### Categorisation of monogenic conditions

Monogenic indications within the PGT-M cohort were coded according to certain disease characteristics. The severity of monogenic conditions was scored using the disease severity classification system published by Lazarin et al. in 2014 [24]. This is a validated algorithm for the objective classification of monogenic disease severity. Following the application of the algorithm, each condition was assigned to one of four severity categorises — profound, severe, moderate, and mild.

Monogenic conditions were then coded according to availability of treatment, inheritance pattern, the age of onset of the condition and the utility of prenatal screening techniques in condition diagnosis (including capacity for diagnosis using prenatal ultrasound, or via the detection of sex chromosomes using non-invasive prenatal testing). Codes on inheritance pattern, phenotype, and treatment options were informed by consulting resources including GeneReviews, Online Mendelian Inheritance in Man and Orphanet. If these sources did not provide sufficient information, relevant scientific literature was reviewed.

### Statistical analyses

Following linkage, confirmatory testing rates were calculated using two denominators — uptake of testing per patient and uptake of testing per clinical pregnancy.

The N-1 chi-square test was used to test for differences in confirmatory testing uptake between different phenotypic groups, with the MedCalc Comparisons of Proportions Calculator [25]. Statistical significance was defined as a *p* value < 0.05. 

## Results

During the study period, there were 176 clinical pregnancies following the transfer of a PGT-M tested embryo in 132 Victorian patients. The median maternal age at the time of pregnancy was 32 years (range 25–41). Prenatal diagnosis was performed in 12/176 (8.3% [95% CI: 4.7–14.3%]) pregnancies and 11/132 (6.8% [95% CI: 3.9–11.5%]) patients. In all cases, prenatal diagnostic testing was performed for the monogenic indication alone, with no secondary indication (such as increased probability of aneuploidy) listed. The outcomes of the 176 clinical pregnancies were 154 (87.5%) live births and ongoing pregnancies, 21 (11.9%) losses before 20 weeks gestation, and one (0.6%) stillbirth. The single pregnancy loss that occurred following prenatal diagnosis was a termination of pregnancy due to an unrelated finding in pregnancy. PGT-M clinical outcomes of the two groups are presented in Table 1.

**Table 1:** Clinical outcomes in PGT-M patients with and without confirmatory prenatal diagnosis

During study period	Confirmatory prenatal diagnosis for monogenic condition		No confirmatory prenatal diagnosis for monogenic condition		Total PGT-M cohort
	***N***	**%**	***N***	**%**	***N***
Number of clinical pregnancies	12	7	164	93	176
Number of live births and ongoing pregnancies	11	7	143	93	154
Number of fetuses lost before 20 weeks gestation	1	5	20	95	21
Number of still births	0	0	1	100	1

This table presents the clinical outcomes of pregnancies in a PGT-M cohort, divided between those with and without confirmatory prenatal diagnosis for monogenic conditions. Of the 176 clinical pregnancies, 7% (*n* = 12) had confirmatory prenatal diagnosis, while 93% (*n* = 164) did not. The outcomes include live births and ongoing pregnancies, with 154 across the cohort. Additionally, the number of fetal losses before 20 weeks gestation and stillbirths are also shown, with 21 fetal losses and 1 stillbirth occurring in the cohort

**Fig. 2:**
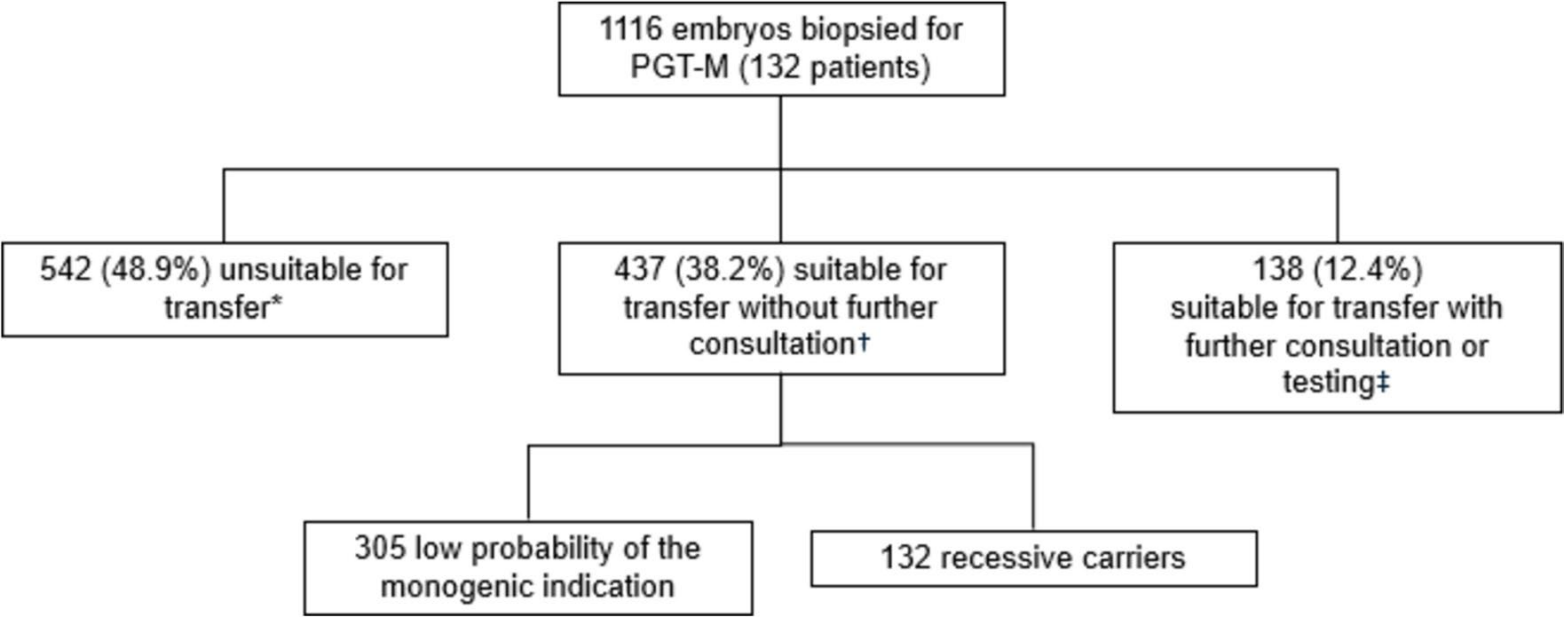
Embryo testing outcomes — suitability for transfer following PGT-M. *Unsuitable for transfer refers to an embryo that is either affected by the condition of interest and/or aneuploid. †Suitable for transfer refers to an embryo that is either low risk for the condition of interest, or a recessive carrier of the condition of interest, and euploid. ‡ Suitable for transfer with further consultation and testing refers includes embryos with an inconclusive result, biopsy performed but testing not yet performed, low-moderate chromosomal mosaicism, or a female carrier of an X-linked dominant condition. These embryos may be considered suitable for transfer following consultation with a specialist, or if additional testing is performed

Among the 132 patients, a total of 1116 embryos were biopsied for PGT-M testing. Of these 437 (38.2%) were suitable for transfer without further consultation. PGT-M testing outcomes are presented in Fig. 2.

**Fig. 3:**
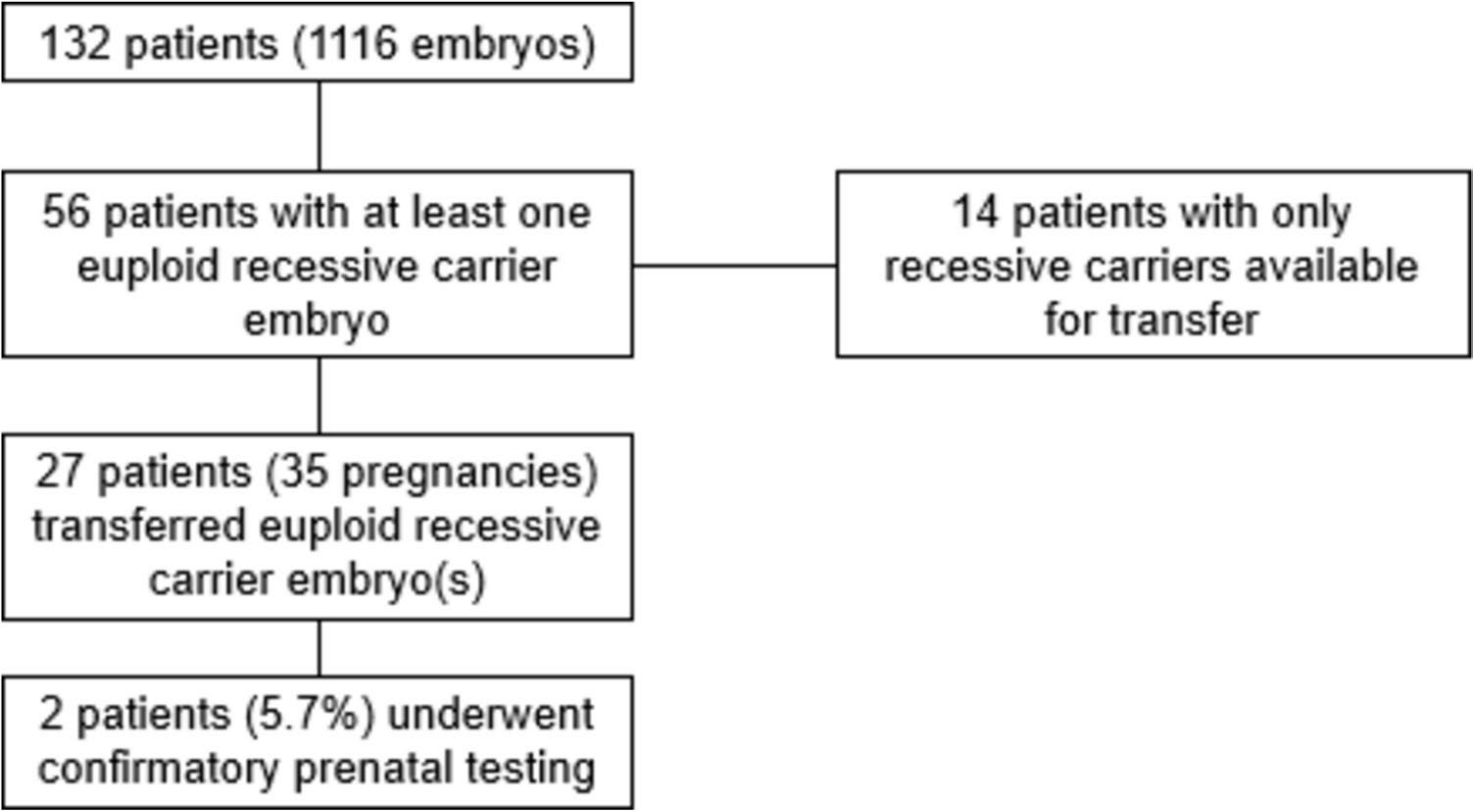
Transfer and prenatal diagnostic testing uptake among individuals with euploid carrier embryos

Of those who transferred a euploid recessive carrier embryo, 2 (5.7%) underwent confirmatory prenatal diagnostic testing. Figure 3 details the number of individuals with a euploid carrier embryo, how many proceeded with its transfer, and their uptake of confirmatory testing.

### Patterns of testing access by disorder categorisation

The 132 patients undertook PGT-M for 68 different conditions. The most common conditions.

were Huntington’s disease (12 patients), Cystic Fibrosis (10 patients), Beta-thalassaemia (9.

patients), and Fragile X (5 patients). During the study period, 9 patients accessed exclusion testing for autosomal dominant indications. PGT-M for the remaining 64 conditions were accessed by 1–4 patients each. These conditions are not reported due to potential patient privacy concerns. Disease characteristics of the 68 conditions are presented in Table 2. Among the conditions studied, the most frequently observed characteristics were moderate severity (63.2%), autosomal dominant inheritance (48.5%), absence of a detectable phenotype on prenatal ultrasound (67.6%), availability of supportive treatment only (82.4%), non-sex linked inheritance, making NIPT unsuitable as a method for prenatal screening (77.9%), symptom onset before adulthood (76.5%) and non-lethal in infancy (91.2%).

**Table 2:** Characteristics of conditions for which PGT-M was undertaken

Category	Number of monogenetic conditions (total = 68)	%
**Condition severity classification**		
Mild	3	4.4
Moderate	42	61.7
Severe	17	25.0
Profound	5	7.4
**Inheritance pattern**		
Autosomal recessive	21	30.9
Autosomal dominant	33	48.5
X-linked recessive	10	14.7
X-linked dominant	5	7.4
**Prenatal ultrasound phenotype**		
No	46	67.6
Yes/sometimes	22	32.4
**Treatment availability**		
Supportive treatment only	56	82.4
Effective treatment, onerous	3	4.4
Effective treatment, difficult to access (e.g. transplantation)	2	2.9
Preventative measures and surveillance	7	10.3
**Utility of NIPT as a prenatal screening technique**		
No	53	77.9
Yes	15	22.1
**Onset of symptoms in adulthood**		
No	52	76.5
Yes	15	22.1
Variable onset	1	1.5
**Lethal in infancy**		
No	62	91.2
Yes	6	8.8

This table categorises 68 monogenic conditions screened in the PGT-M cohort based on several clinical and genetic factors. Conditions are classified by severity, with 61.7% classified as moderate, 25% as severe, and 7.4% as profound. The inheritance patterns include 48.5% autosomal dominant, 30.9% autosomal recessive, and 14.7% X-linked recessive. Prenatal ultrasound phenotypes were absent for 67.6% of conditions, while 32.4% were associated with detectable phenotypes. Most conditions (82.4%) had only supportive treatments available, while 10.3% benefit from preventative measures or surveillance. The utility of NIPT for screening was low, with non-sex linked inheritance in 91.2% of conditions, making NIPT for the detection of sex chromosomes unsuitable as a method for prenatal screening in most cases. Additionally, 22.1% had onset of symptoms in adulthood, and 8.8% were lethal in infancy

A comparison of characteristics between individuals who undertook confirmatory prenatal diagnostic testing versus those who did not is presented in Table 3.

**Table 3:** Disease characteristics in the prenatal diagnosis and no prenatal diagnosis groups. The value in bold indicates statistical significance

Category	Prenatal diagnosis, *n* = 11		No prenatal diagnosis, *n* = 121		*P*
	***N***	**%**	***N***	**%**	
**Condition severity classification**					
Mild	0	0	3	2.5	*P* = 0.6
Moderate	7	63.6	88	72.7	*P* = 0.5
Severe	2	18.2	25	20.7	*P* = 0.8
Profound	2	18.2	5	4.1	***P* = 0.05**
**Inheritance pattern**					
Autosomal recessive	4	36.4	38	31.4	*P* = 0.8
Autosomal dominant	5	45.5	56	46.3	*P* = 0.9
X-linked recessive	2	18.2	15	12.4	*P* = 0.7
X-linked dominant	0	0	12	9.9	*P* = 0.3
**Prenatal ultrasound phenotype**					
No	7	63.6	90	74.4	*P* = 0.95
Yes/sometimes	4	36.4	31	25.6	*P* = 0.95
**Treatment availability**					
Supportive treatment only	11	100	107	88.4	*P* = 0.2
Effective treatment, onerous	0	0	0	0	-
Effective treatment, difficult to access (e.g. transplantation)	0	0	5	4.1	*P* = 0.6
Preventative measures and surveillance	0	0	9	7.4	*P* = 0.3
**Utility of NIPT as a prenatal screening technique**					
No	9	81.8	95	78.5	*P* = 0.8
Yes	2	18.2	26	21.5	*P* = 0.8
**Onset of symptoms in adulthood**					
No	10	90.9	83	68.6	*P* = 0.1
Yes	1	9.1	36	29.8	*P* = 0.1
Variable onset	0	0	2	1.7	*P* = 0.7
**Lethal in infancy**					
No	11	100	113	93.4	*P* = 0.4
Yes	0	0	8	6.6	*P* = 0.4

This table presents the distribution of condition severity, inheritance patterns, prenatal ultrasound phenotypes, treatment availability, and screening utility between the prenatal diagnosis (*n* = 11) and no prenatal diagnosis (*n* = 121) groups. Both groups show similar patterns in moderate and severe conditions, but the prenatal diagnosis group has a higher proportion of profound conditions (18.2% vs 4.1%, *P* = 0.05). Inheritance patterns and prenatal ultrasound findings are comparable between the groups, as are the availability of supportive treatments. There is no significant difference in the utility of NIPT or the presence of adult-onset or lethal-in-infancy conditions across both groups

The group that undertook confirmatory testing had a significantly higher proportion of profoundly severe monogenic disease (18.2% vs 4.2%, *p* = 0.046). The group that did not undertake confirmatory testing had non-significant trends to higher proportions of conditions with preventative treatment options (7.4% and 0.0%) or adult-onset conditions (30.0% vs 9.1%). 

## Discussion

### Overall findings

We found low rates of prenatal diagnostic testing within our PGT-M cohort, with less than one in ten individuals undergoing confirmatory prenatal diagnosis. This was despite all patients routinely receiving genetic counselling on the limitations of PGT-M and recommendations for follow-up prenatal diagnosis.

While our rate of uptake is similar to reported rates for confirmatory testing after PGT-A, it is lower than previous reports of PGT-M cohorts [7]. Differences in uptake may be due to variations in PGT-M and prenatal care practice across clinics, including counselling surrounding PGT-M and its limitations, and differences in PGT-M protocols such as concurrent aneuploidy screening and test accuracy, and differences in accessibility including cost of treatment. In the 2022 study by Bunnell et al., 44% of patients did not have aneuploidy screening performed on their embryos prior to transfer [10]. In contrast, all embryos in our cohort were concurrently screened for aneuploidy. It is possible our cohort were therefore less likely to have another indication for testing, such as a high probability aneuploidy screening result, or due to ultrasound anomaly. In contrast, Toft et al.’s 2022 study found that most respondents who undertook CVS following PGT-M cited clinical recommendations as a motivating factor. This may indicate this cohort received different counselling on the limitations of PGT-M compared to our cohort, possibly influencing their decision to pursue confirmatory testing.

Although the current study did not collect data on patient decision making, previous research suggests reasons for declining confirmatory prenatal testing include concern regarding procedure-related miscarriage, feeling as though testing outcomes would not alter pregnancy decision-making, and misunderstanding of testing recommendations [11, 26, 27]. Additionally, patients may weigh the residual risk of misdiagnosis against the risk of confirmatory testing.

Low uptake may reflect low patient acceptance of existing guidelines. While existing guidelines were primarily developed to facilitate informed consent, the recommendation for prenatal diagnosis may also be perceived by patients as a defensive practice to protect providers from litigation. It is crucial that clinical guidelines are patient-centred, rather than developed due to medico-legal concerns [28]. Incorporating patient preferences into the development of clinical guidelines facilitates shared decision-making, enables informed consent, and builds patient engagement [29, 30]. This approach supports effective healthcare delivery and promotes patient autonomy, aligning with ethical practice.

Three trends were noted during our analysis. Among those that undertook prenatal diagnostic testing, there was (i) a greater proportion of conditions with profound severity; (ii) there were a smaller proportion of conditions that were adult onset; and (iii) a smaller proportion of conditions amenable to treatment. While these trends were not statistically significant, due to the small size of each subgroup, each trend is clinically plausible and is consistent with our clinical experiences of patient decision-making.

These trends may be explained by various factors which influence an individual’s decision to undertake prenatal diagnostic testing. Individuals may confirm the genetic status of their pregnancy to gain reassurance, prepare for the birth of a child with a genetic condition, or to inform pregnancy outcomes, such as termination of pregnancy. When considering pregnancy outcomes, patients often reflect on the quality of life of a child with the genetic indication [31].

A 2013 study exploring reasons for termination in the setting of fetal anomalies reported that for many pregnant people, the emotional trauma of delivery a baby with poor quality of life dictated their decision-making [12]. It is possible that individuals feel inclined to confirm conditions of profound severity, as this information is more likely to inform pregnancy outcomes. Perceived severity is a key dimension in the Health Belief model, a framework used to explain health behaviour acceptance [32]. Within this model, patients are more likely to undertake a health behaviour to avoid conditions they perceive to be severe [33]. Extending from this, it is possible patients feel a greater need to confirm the accuracy of PGT-M in the context of conditions with greater severity.

Consideration of quality of life may also contribute to the lower testing uptake observed for conditions that are adult onset or have preventative treatment options available. While adult-onset conditions can greatly affect the quality of life in later years, there is the possibility of good quality of life preceding condition onset [34]. This may make individuals feel more comfortable utilising PGT-M as a risk reduction approach alone, and patients may feel less concerned with receiving diagnostic confirmation of the prenatal genetic status [35]. Additionally, individuals may feel more comfortable utilising PGT-M to reduce reproductive risk for conditions where preventative treatment options are available, as these can drastically improve prognostic outcomes and subsequent quality of life of affected individuals [36]. The observed low testing uptake for adult-onset conditions and those with preventative treatment options may reflect these perceptions.

Prenatal diagnosis for adult-onset conditions can raise unique ethical concerns. As highlighted earlier, termination of pregnancy may not feel appropriate due to perceived quality of life prior to condition onset. However, if testing is conducted without the intention to terminate in the context of a positive result, it may disregard the autonomy of the future individual (the ‘right not to know’ one’s genetic status) [34]. Individuals may also be hopeful that future treatment options could alter the prognosis of adult-onset conditions [36, 37]. These ethical considerations and optimism for future treatment options may also contribute to the lower testing uptake observed for adult-onset conditions.

The current study is limited by the small size of the prenatal diagnosis group, which limited the statistical significance of our subgroup analysis. This analysis is therefore exploratory, identifying potential trends to guide future research directions. Further investigation through larger quantitative studies and qualitative research will be valuable to explore possible associations between condition characteristics and confirmatory testing uptake. Our study is also limited by being a single-centre study. While our fertility centre is one of the state’s largest PGT-M providers, there may be variation in uptake rate across different fertility centres that is missed by the current study. Additionally, we were not able to measure the PGT-M misdiagnosis rate in our cohort due to the unavailability of data on postnatal outcomes. However, as the primary aim was to analyse patient uptake of prenatal diagnosis rather than evaluate the accuracy of PGT-M, this limitation did not affect our ability to answer the research question. Finally, as we did not collect information on patient decision-making, we are only able to speculate on reasons for the observed low uptake.

This study contributes important data to an underresearched area. The study’s strengths include the consistent use of karyomapping over the study period and the consistent provision of genetic counselling, including discussion surrounding the limitations of testing, to all patients. This reflects current laboratory practice and enables concurrent aneuploidy screening on all embryos transferred during the study period. This reduces the chance of prenatal diagnosis undertaken due to aneuploidy confounding results. Our study is also strengthened by using a state-wide dataset for linkage. 

### Future research

Our results demonstrate low prenatal diagnosis uptake in our PGT-M population. Further research using patient-reported outcomes are needed to understand why most individuals do not follow recommendations to have prenatal diagnostic testing in a PGT-M conception. Qualitative research exploring the decision-making process is needed to understand patient perceptions, preferences, and experience, and could be used to improve genetic counselling of this indication group. Future research should also explore patient acceptance of existing guidelines. 

## Conclusion

The majority of individuals who conceive following the transfer of a PGT-M-tested embryo do not undertake confirmatory prenatal diagnostic testing. The low uptake of prenatal diagnosis raises important considerations for genetic counselling and patient acceptability of current clinical recommendations.

## Data Availability

The data underlying this article are not publicly available due to the conditions of human research ethics approval. Non-identifiable participant data from this study are available to researchers affiliated with a recognised academic institution, upon reasonable request to the corresponding author. The study investigators may contribute aggregate and non-identifiable individual patient data to researchers whose proposed use of the data has been ethically reviewed and approved by an independent committee and following signing of an appropriate research collaboration agreement.
